# The impact of mass gatherings and holiday traveling on the course of an influenza pandemic: a computational model

**DOI:** 10.1186/1471-2458-10-778

**Published:** 2010-12-21

**Authors:** Pengyi Shi, Pinar Keskinocak, Julie L Swann, Bruce Y Lee

**Affiliations:** 1 Stewart School of Industrial and Systems Engineering, Georgia Institute of Technology, 755 Ferst Drive, Atlanta, Georgia, USA; 2 Medicine, Epidemiology, and Biomedical Informatics, School of Medicine and Graduate School of Public Health, University of Pittsburgh, 200 Meyran Ave., Suite 200, Pittsburgh, Pennsylvania, USA

## Abstract

**Background:**

During the 2009 H1N1 influenza pandemic, concerns arose about the potential negative effects of mass public gatherings and travel on the course of the pandemic. Better understanding the potential effects of temporal changes in social mixing patterns could help public officials determine if and when to cancel large public gatherings or enforce regional travel restrictions, advisories, or surveillance during an epidemic.

**Methods:**

We develop a computer simulation model using detailed data from the state of Georgia to explore how various changes in social mixing and contact patterns, representing mass gatherings and holiday traveling, may affect the course of an influenza pandemic. Various scenarios with different combinations of the length of the mass gatherings or traveling period (range: 0.5 to 5 days), the proportion of the population attending the mass gathering events or on travel (range: 1% to 50%), and the initial reproduction numbers R_0 _(1.3, 1.5, 1.8) are explored.

**Results:**

Mass gatherings that occur within 10 days before the epidemic peak can result in as high as a 10% relative increase in the peak prevalence and the total attack rate, and may have even worse impacts on local communities and travelers' families. Holiday traveling can lead to a second epidemic peak under certain scenarios. Conversely, mass traveling or gatherings may have little effect when occurring much earlier or later than the epidemic peak, e.g., more than 40 days earlier or 20 days later than the peak when the initial R_0 _= 1.5.

**Conclusions:**

Our results suggest that monitoring, postponing, or cancelling large public gatherings may be warranted close to the epidemic peak but not earlier or later during the epidemic. Influenza activity should also be closely monitored for a potential second peak if holiday traveling occurs when prevalence is high.

## Background

During the 2009 H1N1 influenza pandemic, concerns arose about the potential negative effects of mass public gatherings and travel on the course of the pandemic. The World Health Organization (WHO), the U.S. Centers for Disease Control and Prevention (CDC), and many other public health organizations published recommendations [1-8] suggesting the public defer non-essential travel to infected areas and emphasizing taking appropriate precautions (e.g., hand hygiene) during traveling, attending and/or hosting mass gathering events. However, the decisions regarding cancelling or postponing mass gatherings are left to local authorities; travel restrictions are generally not recommended [4,9-13], but some countries have introduced new travel regulations relating to the 2009 H1N1 outbreaks [14]. Later in December 2009, when the pandemic appeared to be subsiding, public health officials contemplated whether changes in social mixing patterns due to a combination of Holiday travel with school and workplace closures could lead to a subsequent surge of cases ("a third wave") similar to those seen in 1918 and 1957 [15,16].

Previous studies have shown the effects of social mixing patterns and distancing measures (such as school closures and travel restrictions) on the spread of infectious diseases and epidemics [17-26]. A recent study showed how viral mutation can lead to an additional epidemic peak [27]. However, few studies have explored the potential negative impact of public gatherings and Holiday travel during an epidemic.

Better understanding the potential effects of changes in social mixing patterns could help public officials determine if and when to cancel large public gatherings or enforce regional travel restrictions, advisories, or surveillance during an influenza pandemic. Therefore, we developed a computer simulation model using detailed data from the state of Georgia to explore how various changes in social mixing and contact patterns, representing mass gatherings and Holiday traveling, may affect the course of an influenza pandemic.

## Methods

**Figure 1:**
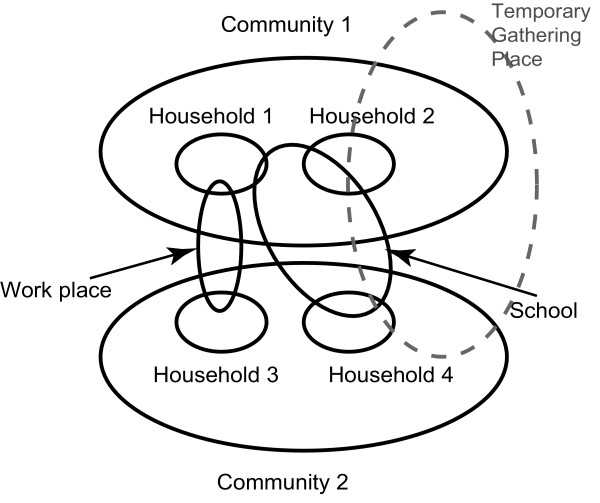
**An example of the contact network**. The figure shows an example of the contact network, i.e., how persons interact with each other in households, workplaces, schools, communities, and/or temporary mass gathering locations.

Our study utilizes a previously-described spatially and temporally explicit agent-based simulation model of the state of Georgia that consists of a population of computer agents, with each agent representing an individual programmed with socio-demographic characteristics and behaviors [27]. Each agent has an assigned household according to distributions from the 2000 U.S. Census Data [28]. Agents interact with each other in homes, peer groups (workplaces and schools), communities, and/or during mass gathering events and Holiday traveling [11,29]. The model population consists of five age groups: 0-5, 6-11, 12-18, 19-64, and ≥65 years. Table 1 lists the distributions of the size of the households, peer groups and communities. Figure 1 diagrams the social network for the model. Each day individuals move among different locations and homogenously mix within those locations.

**Table 1:** Key Model Parameters

Parameter	Description	Baseline Values	Reference
p_A_	Probability of infected individual remaining asymptomatic throughout course of infection	0.4 for working adults, 0.25 for others	[29,30,49,52]

p_H_	Probability of symptomatic individual requiring hospitalization	0.18 for ages 0-5, 0.06 for ages 6-64, 0.12 for ages 65+	[29,30]

p_D_	Probability of hospitalized individual not surviving	0.344 for ages 0-5, 0.172 for ages 6+	[30,53]

Duration of E+ I_P_	Duration of exposed and presymptomatic stages	Weibull distribution with mean 1.48 and standard deviation 0.47, and offset 0.5	[30,39]

Duration of I_P_	Duration of presymptomatic stage	0.5 (constant)	[30,39]

Duration of I_S_	Duration of symptomatic stage	Exponential distribution with mean 2.7313 (mean = 7 in the sensitivity analysis)	[30,41,42]

Duration of I_A_	Duration of asymptomatic stage	Exponential distribution with mean 1.63878 (mean = 7 in the sensitivity analysis)	[30,41,42]

Duration of I_H_	Duration of hospitalization	Exponential distribution with mean 14	[30,39]

Household Size	Number of individuals in each household	1 person: 10.33%; 2 persons 23.55%; 3 persons: 20.45%; 4 persons: 23.00%; 5 persons: 12.79%; 6 persons: 5.91%; 7 persons: 3.97%.	[28]

School Classroom Size	Number of individuals in each classroom	Uniform distribution (9,19) for ages 0-5; uniform distribution (15,25) for ages 6-11; and uniform distribution (25,35) for ages 12-18	[30,31]

Workplace Size	Number of individuals in each workplace	Poisson distribution with mean 20 (maximum 1000)	[29,30]

Community Size	Number of people in each census tract (1615 census tracts in the state of Georgia)	Maximum = 29341, minimum = 218	[28]

p%	Proportion of the population that attends mass gatherings or travels during the experiments	1%, 5%, 10%, and 25% for the non-Holiday scenarios; 25% and 50% for the Holiday scenarios	[32-37,43,44]

Initial R_0_	Reproductive rate (average number of secondary cases generated by each infected individual) for each experiment before social mixing changes are introduced	1.3, 1.5, and 1.8	[11,20,29,30,39]

Resulting R_0_	Reproductive rate (average number of secondary cases generated by each infected individual) for each experiment after social mixing changes are introduced	See Tables 2-4	

θ	Proportion of transmissions that occur at presymptomatic/asymptomatic stage	0.3	[30]

ω	Proportion of infections generated by individuals who are asymptomatic	0.15	[30]

γ	Proportion of transmissions that occur outside the households	0.7	[11]

δ	Proportion of transmissions outside the home that occur in the community	0.5	[11]

The table shows the explanations, values and sources for the key parameters we used in the simulation model.

At the beginning of each simulation run, all agents in the population are susceptible. On Day 1, three infected agents are introduced into the population. Contact with an infectious agent has a probability of transmission of the virus to the susceptible agent. A newly infected agent then progresses through the following stages: Susceptible-Exposed-Infected-Recovered (SEIR), based on the incubation and infectious periods of the disease [30]. After being infected, each individual first progresses through the incubation period, then through the presymptomatic phase, and then has a probability *p_A _*of remaining asymptomatic and a probability (*1-p_A_*) of becoming symptomatic during the infectious period. Each symptomatic individual has a probability *p_H _*of requiring hospitalization (H). Each hospitalized individual has a probability *p_D _*of dying. Individuals who survive infection eventually assume the recovered state and are immune to infection. Development of the disease and contact model is based on methods used by Ferguson et al. and Wu et al. [11,30]. Table 1 lists the values and sources of key parameters.

### Mass Social Mixing: Large Public Gatherings and Holiday Travel

To explore the effects of mass social mixing changes (e.g., large public gatherings and Holiday traveling), we divide the year into a "regular" period and a "traveling" (or "mass gathering") period. The "traveling" period starts at day t* after the introduction of the initial infected case and lasts for *l *days; the remaining days before and after this "traveling period" comprise the "regular" period.

During the regular period, agents move back and forth between households and workplaces or schools [27,31]. They mix in the workplaces or schools during the day and in their households during the night. Agents also mix in the communities during the day and night by visiting common areas such as grocery stores, churches, theaters, etc.

At the beginning of the traveling period, we select *p% *of the total agent population (in two different ways, see below) to change mixing patterns. They mix in a large group (i.e., "traveling/mass gathering group") to model temporal mass gathering locations/events, e.g., airports, shopping malls, or the annual Georgia Tech versus University of Georgia football game. We consider the following two scenarios:

1. Non-Holiday: *p% *of the total agent population is sampled randomly. Agents selected to mix in the "traveling/mass gathering group" only have contact with each other in the group, and no longer interact with their family members or classmates/colleagues, or mix in their usual communities. The *(1-p)% *agents not in the traveling group retain their usual mixing routines, e.g., mix in their workplaces or schools during the day and in their households during the night. This scenario represents mass public gatherings, e.g., a football game, road race, concert, convention, or demonstration [32-37], where one does not necessarily attend the events or travel with his/her family. The traveling/gathering group can include event attendees, visitors, and local residents.

2. Holiday: A subset of households is randomly sampled so that *p% *of the total agent population is chosen to mix in the "traveling/mass gathering group." The agents travel with their family members (i.e., mix in the household day and night), and also interact with other agents in the traveling group during the day. However, they no longer mix in their schools, workplaces or usual communities. The agents not selected for travel reduce their peer group mixing activities [21,22]. Schools and a percentage of workplaces (baseline 50%) are closed during the traveling period (*l *days) so that agents no longer mix in these locations. This setting represents travel or mass gatherings during a holiday, e.g., Thanksgiving or New Year's Eve.

When the traveling period ends, all the agents return to their regular mixing routines.

The calibration procedure involves several steps. First, we establish the social network group sizes (i.e., households, workplaces, and schools) based on data listed in Table 1, and we assume homogeneous mixing within each group. Then, studies of previous pandemics provide the correlation between R_0 _and the resulting attack rate. Therefore, for a given R_0_, we target the corresponding attack rate, i.e., adjust transmission parameters until the appropriate attack rate is obtained. This method has been used in numerous previous studies [11,17,30,38-42]. Additional details on the transmission models and the calculation of the parameters during the regular period and the traveling period are available in [Additional file 1].

### Simulation Runs and Sensitivity Analyses

To study the impact of traveling and mass gathering events on the course of an influenza pandemic, we test different scenarios with three initial influenza reproductive rates (the initial R_0 _before any social mixing changes occurred): 1.3, 1.5, and 1.8 (see [Additional file 1] for more details), which correspond to R_0 _estimates from past pandemics in 1918, 1957, 1968, and 2009 [11,20,29,30,39]. Separate scenarios also explore the effects of using different "traveling/gathering" starting dates t* (Day 30, 60, 90, 120, 180), "traveling/gathering" durations *l *(0.5 day, 1 day, 2 days, and 3 days for the non-Holiday scenario, 3 and 5 days for the Holiday scenario), and the proportion of the population that travels/gathers during this period *p *(1%, 5%, 10% and 25% for the non-Holiday scenario [32-37], 25% and 50% for the Holiday scenario [43,44]).

To study the regional impact of traveling and mass gathering events, we explore various proportions for the population who participate in traveling/gathering (i.e., different *p *values) in different locations. For example, similar to the Annual Cherry Blossom Festival in Macon, Georgia, we assume in one experimental scenario that 50% of the population travels/gathers in Bibb County and its nearest 5 counties [28], and 9.5% of the population travels/gathers in other counties (so that for the entire population *p *= 10%) under the non-Holiday setting.

The total number of experimental scenarios is 125 for the non-Holiday scenario and 60 for the Holiday scenario with 10 replications for each experiment unless indicated otherwise. The time horizon for each replication is 365 days.

## Results

For the non-Holiday scenario, we focus on the characteristics of peak prevalence and the total attack since only one epidemic peak appears; for the Holiday scenarios, we focus on whether two epidemic peaks are present (i.e., the influenza activity declines first and increases later). In the non-Holiday setting, we also examine the impact of transmissions to the traveler and their family and within regions where gathering occurs. Tables 2, 3, 4 report the initial baseline R_0 _(before social mixing changes are introduced) values, the peak prevalence, the total attack rate, and the resulting R_0 _values after the mass social mixing changes were instituted for experiments under the non-Holiday setting.

**Table 2:** Results from Different Mass Gathering Scenarios (Initial R0 = 1.5)

% population traveling (*p*)	Traveling Period Start Duration		Resulting R_0_	Peak Prevalence	Peak Day	Total Attack Rate
*p *= 1%	Day 30	0.5	1.50	2.73%	70	51.0%
	
	Day 30	1	1.50	2.76%	70	51.0%
	
	Day 30	2	1.50	2.78%	71	51.0%
	
	Day 30	3	1.50	2.79%	70	51.0%
	
	Day 60	0.5	1.50	2.74%	70	51.0%
	
	Day 60	1	1.50	2.76%	70	51.0%
	
	Day 60	2	1.50	2.74%	70	51.0%
	
	Day 60	3	1.50	2.75%	71	51.0%

*p *= 5%	Day 30	0.5	1.50	2.74%	69	51.0%
	
	Day 30	1	1.50	2.77%	70	51.0%
	
	Day 30	2	1.50	2.77%	70	51.0%
	
	Day 30	3	1.50	2.80%	70	51.0%
	
	Day 60	0.5	1.50	2.74%	69	51.0%
	
	Day 60	1	1.50	2.81%	70	51.2%
	
	Day 60	2	1.51	2.83%	70	51.2%
	
	Day 60	3	1.50	2.78%	70	51.1%

*p *= 10%	Day 30	0.5	1.50	2.74%	69	51.0%
	
	Day 30	1	1.50	2.78%	69	51.0%
	
	Day 30	2	1.50	2.80%	69	51.0%
	
	Day 30	3	1.50	2.82%	68	51.1%
	
	Day60	0.5	1.50	2.80%	69	51.0%
	
	Day 60	1	1.51	2.85%	70	51.3%
	
	Day 60	2	1.51	2.89%	69	51.4%
	
	Day 60	3	1.50	2.80%	70	51.1%

*p *= 25%	Day 30	0.5	1.50	2.79%	69	51.0%
	
	Day 30	1	1.50	2.80%	68	51.1%
	
	Day 30	2	1.50	2.80%	68	51.1%
	
	Day 30	3	1.50	2.83%	70	51.0%
	
	Day 60	0.5	1.51	2.90%	69	51.4%
	
	Day 60	1	1.52	3.04%	69	51.7%
	
	Day 60	2	1.53	3.12%	69	52.0%
	
	Day 60	3	1.51	2.96%	71	51.4%

Baseline			1.50	2.73%	70	51.0%

The table shows the total attack rate (i.e., proportion of population that has ever been infected), the peak prevalence day and value in the non-Holiday scenarios, with several combinations of values for *l *(duration of the traveling/mass traveling period) and *p *(the proportion of the population traveling/gathering) when the initial R_0 _equals to 1.5. The resulting R_0 _values (after adding the traveling/mass gathering period) are obtained from the baseline scenarios (without traveling/gathering) to match the peak prevalence and the total attack rate showed in this table. The standard deviation is 0.04-0.09% for the peak prevalence and is 0.17-0.30% for the total attack rate.

**Table 3:** Results from Different Mass Gathering Scenarios (Initial R0 = 1.3)

% population traveling (*p*)	Traveling Period Start Duration		Resulting R_0_	Peak Prevalence	Peak Day	Total Attack Rate
*p *= 1%	Day 60	0.5	1.30	0.96%	98	32.5%
	
	Day 60	1	1.30	0.97%	99	32.5%
	
	Day 60	2	1.30	0.96%	98	32.8%
	
	Day 60	3	1.30	0.98%	98	32.9%
	
	Day 90	0.5	1.30	0.96%	97	32.5%
	
	Day 90	1	1.30	0.97%	98	32.6%
	
	Day 90	2	1.30	0.98%	97	32.8%
	
	Day 90	3	1.30	0.98%	97	32.8%

*p *= 5%	Day 60	0.5	1.30	0.97%	96	32.6%
	
	Day 60	1	1.30	0.98%	97	32.6%
	
	Day 60	2	1.30	0.98%	97	32.8%
	
	Day 60	3	1.30	1.00%	96	32.9%
	
	Day 90	0.5	1.30	0.98%	97	32.8%
	
	Day 90	1	1.30	0.98%	96	32.7%
	
	Day 90	2	1.31	1.00%	99	33.1%
	
	Day 90	3	1.30	0.97%	101	32.7%

*p *= 10%	Day 60	0.5	1.30	0.98%	96	32.8%
	
	Day 60	1	1.30	0.98%	96	32.7%
	
	Day 60	2	1.30	0.99%	95	32.9%
	
	Day 60	3	1.30	1.01%	97	32.9%
	
	Day 90	0.5	1.30	0.99%	98	32.8%
	
	Day 90	1	1.31	1.00%	97	33.1%
	
	Day 90	2	1.31	1.02%	99	33.1%
	
	Day 90	3	1.30	0.98%	99	32.8%

*p *= 25%	Day 60	0.5	1.30	0.98%	97	32.8%
	
	Day 60	1	1.30	1.00%	97	32.7%
	
	Day 60	2	1.31	1.05%	94	33.2%
	
	Day 60	3	1.30	1.03%	99	32.7%
	
	Day 90	0.5	1.31	1.04%	98	33.1%
	
	Day 90	1	1.31	1.07%	99	33.3%
	
	Day 90	2	1.32	1.11%	99	33.7%
	
	Day 90	3	1.31	1.02%	99	33.0%

Baseline			1.30	0.96%	94	32.4%

The table shows the total attack rate (i.e., proportion of population that has ever been infected), the peak prevalence day and value in the non-Holiday scenarios, with several combinations of values for *l *(duration of the traveling/mass traveling period) and *p *(the proportion of the population traveling/gathering) when the initial R_0 _equals to 1.3. The resulting R_0 _values (after adding the traveling/mass gathering period) are obtained from the baseline scenarios (without traveling/gathering) to match the peak prevalence and the total attack rate showed in this table. The standard deviation is 0.02-0.05% for the peak prevalence and is 0.22-0.41% for the total attack rate.

**Table 4:** Results from Different Mass Gathering Scenarios (Initial R0 = 1.8)

% population traveling (*p*)	Traveling Period Start Duration		Resulting R_0_	Peak Prevalence	Peak Day	Total Attack Rate
	Start	Duration				
*p *= 1%	Day 30	0.5	1.80	5.99%	50	68.4%
	
	Day 30	1	1.80	5.99%	51	68.4%
	
	Day 30	2	1.80	6.00%	50	68.4%
	
	Day 30	3	1.80	6.00%	50	68.4%
	
	Day 45	0.5	1.80	5.99%	51	68.4%
	
	Day 45	1	1.80	6.00%	51	68.4%
	
	Day 45	2	1.80	5.99%	51	68.4%
	
	Day 45	3	1.80	5.96%	51	68.4%

*p *= 5%	Day 30	0.5	1.80	6.01%	50	68.4%
	
	Day 30	1	1.80	6.01%	50	68.4%
	
	Day 30	2	1.80	6.03%	50	68.4%
	
	Day 30	3	1.80	6.10%	51	68.4%
	
	Day 45	0.5	1.80	6.04%	51	68.4%
	
	Day 45	1	1.80	6.05%	50	68.6%
	
	Day 45	2	1.81	6.09%	51	68.7%
	
	Day 45	3	1.80	5.94%	51	68.4%

*p *= 10%	Day 30	0.5	1.80	6.03%	51	68.4%
	
	Day 30	1	1.80	6.08%	50	68.4%
	
	Day 30	2	1.80	6.12%	50	68.4%
	
	Day 30	3	1.80	6.17%	51	68.4%
	
	Day 45	0.5	1.80	6.05%	51	68.5%
	
	Day 45	1	1.81	6.20%	50	68.6%
	
	Day 45	2	1.80	6.07%	51	68.5%
	
	Day 45	3	1.80	5.99%	51	68.4%

*p *= 25%	Day 30	0.5	1.80	6.08%	50	68.4%
	
	Day 30	1	1.81	6.16%	50	68.5%
	
	Day 30	2	1.82	6.31%	50	68.6%
	
	Day 30	3	1.82	6.40%	50	68.5%
	
	Day 45	0.5	1.82	6.20%	51	68.8%
	
	Day 45	1	1.83	6.49%	50	69.3%
	
	Day 45	2	1.83	6.58%	51	69.5%
	
	Day 45	3	1.82	6.21%	53	68.6%

Baseline			1.80	5.99%	50	68.4%

The table shows the total attack rate (i.e., proportion of population that has ever been infected), the peak prevalence day and value in the non-Holiday scenarios, with several combinations of values for *l *(duration of the traveling/mass traveling period) and *p *(the proportion of the population traveling/gathering) when the initial R_0 _equals to 1.8. The resulting R_0 _values (after adding the traveling/mass gathering period) are obtained from the baseline scenarios (without traveling/gathering) to match the peak prevalence and the total attack rate showed in this table. The standard deviation is 0.08-0.15% for the peak prevalence and is 0.07-0.18% for the total attack rate.

### The Timing of Mass Travel/Public Gatherings t*

As the results from simulating non-Holiday scenarios demonstrate, when the initial R_0 _= 1.5, mass traveling or public gatherings that commence more than 20 days (e.g., t* = 90, 120, or 180) after the epidemic peak (Day 70 in the baseline scenario with R_0 _= 1.5) had little impact on the peak prevalence or the total attack rate. Mass traveling or public gatherings that commence well prior (i.e., more than 40 days) to the epidemic peak (e.g., t* = 30) have a minor but not significant impact, e.g., having 25% of the population traveling increases the peak prevalence from 2.73% (baseline) to 2.80% (around 2% relative increase in the peak percentage) but does not affect the overall attack rate much.

However, mass traveling that begins shortly before the peak prevalence day (e.g., t* = 60, 10 days before the peak in the baseline case) can significantly increase the peak prevalence, e.g., 25% of the population traveling for 1 day increases the peak prevalence from 2.73% (baseline) to 3.04% (around a 11% relative increase) and increases the overall attack rate from 51.0% (baseline) to 51.7%. This translates to an additional 63,502 individuals being infected in Georgia [28]. Table 2, 3, 4 show how different values of t* (the starting time of the "traveling/gathering" period) affect the epidemic under non-Holiday conditions for all the initial R_0 _values we tested.

The results of simulating the Holiday setting show that similar observations hold in that setting. When the initial R_0 _= 1.5, mass traveling/gatherings that occur more than 20 days after the epidemic peak or more than 40 days before the peak do not lead to a second epidemic peak; otherwise, two explicit epidemic peaks can appear under certain scenarios as we demonstrate in the next section.

### Impact of Holiday Traveling on Multiple Peaks

**Figure 2:**
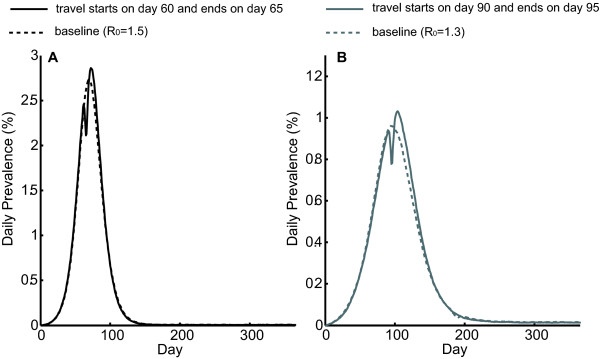
**Epidemic curves in the Holiday scenarios**. The figure shows the daily prevalence of infection (i.e., proportion of the symptomatic and asymptomatic persons over the entire population) for the entire state of Georgia under the Holiday setting. Here 25% of the population travels during a 5-day traveling or mass gathering period with two initial R_0 _values: A) R_0 _= 1.5; B) R_0 _= 1.3.

Figure 2 shows the resulting epidemic curves (i.e., the daily prevalence of infected persons) for the entire state of Georgia under the Holiday scenario where 25% of the population mixes in the "traveling group" during a 5-day traveling period. Figure 2(A) shows the scenario with the initial R_0 _= 1.5 when the traveling period starts on Day 60; Figure 2(B) shows the scenario with the initial R_0 _= 1.3 when the traveling period starts on Day 90.

Figure 2 shows that the Holiday scenario can generate two prevalence peaks, while this is not seen in any of the non-Holiday scenarios we tested. Moreover, various scenarios with different initial R_0 _values, traveling durations and proportions of the population on travel can generate two distinct epidemic peaks when Holiday traveling occurs within 5-20 days (depending on the initial R_0 _values) before the prevalence peak day in the baseline (no traveling) scenario. The prevalence, the timings of the two peaks, and the total attack rate depend on the parameter settings in each scenario.

**Figure 3:**
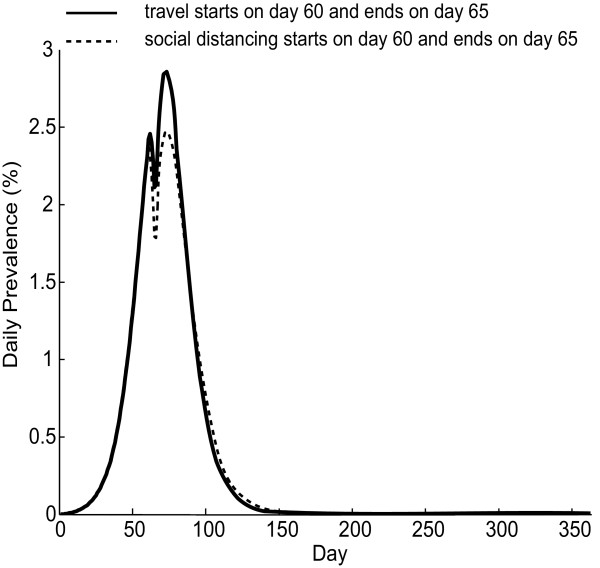
**Epidemic curves in the Holiday and social distancing scenarios**. The figure shows the daily prevalence of infection (i.e., proportion of the symptomatic and asymptomatic persons over the entire population) for the entire state of Georgia under the Holiday and the social distancing settings. Here the initial R_0 _= 1.5; 25% of the population travels during a 5-day period starting on Day 60 (solid curve), or reduces their peer group mixings ("social distancing") during the same period time (dotted curve).

The appearance of the two epidemic peaks is due to partial "social-distancing", as a large proportion of the population no longer mixes in the workplaces/schools when the Holiday (traveling) begins, causing a momentary drop in new cases until the Holiday is over and mixing resumes. To isolate the effects of traveling versus the reduction in peer group mixings, Figure 3 compares the epidemic curves for the entire state of Georgia in the following two scenarios using the initial R_0 _= 1.5: (1) 25% population on travel during a 5-day Holiday period starting on Day 60 as previously described; (2) the same number of persons reduce their peer group mixings and stay at home day and night during a 5-day period starting on Day 60. The second scenario models social distancing or household quarantine. As shown in Figure 3, there are two epidemic peaks in both scenarios; however, the prevalence of the second peak in the social-distancing scenario (2.47%) is lower than that in the traveling scenario (2.86%). The total attack rate in the former is 50.4%, and 51.7% in the latter. This is consistent with our previous observation: traveling/mass gatherings can lead to an increase in the peak prevalence and the total attack rate, but do not cause a second peak alone among the experiments we test.

### The Duration of the Mass Traveling Period (*l*) and the Proportion of the Population Traveling (*p) *under the Non-Holiday Setting

Tables 2, 3, 4 also compare the peak prevalence and the total attack rate in Georgia for different combinations of traveling/gathering duration *l *and the proportion of the population that travels/gathers when the initial R_0 _= 1.3, 1.5, and 1.8 under non-Holiday conditions. As Tables 2, 3, 4 demonstrate, even a half-day event can lead to as high as an 8% increase in the peak prevalence (e.g., with 25% of the population involved in a half-day event starting on Day 90 and the initial R_0 _= 1.3). Moreover, 1-day and 2-day traveling periods result in similar peak prevalence values to each other (a 3% maximum relative difference) and very similar total attack rates (a 1% maximum relative difference).

However, extending the event duration from 2 to 3 days reduces the peak prevalence and total attack rate somewhat (although they remain higher than if mass gathering did not occur) in some scenarios. For example, when the initial R_0 _= 1.5 and 10% of the population is involved in a mass gathering event, the resulting peak prevalence and total attack rate are 2.89% and 51.4%, respectively, after a 2-day event starting on Day 60; however, these values are 2.80% and 51.1%, respectively, after a 3-day event starting at the same time. Note that the baseline average infectious period is 3-4 days (see Table 1); sensitivity analyses show results with infectious periods of 7-8 days [41,42]. Under the new assumption, when the initial R_0 _= 1.5, the total attack rate is 49.05% and the peak prevalence is 4.05% in the baseline scenario without traveling/mass gathering. The total attack rate becomes 51.2%, 51.3%, and 51.4% when the traveling period starts at 20 days before the epidemic peak (in the baseline scenario) and lasts for 1, 2 and 3 days, respectively. The peak prevalence becomes 4.56%, 4.58%, and 4.60%, respectively.

The proportion of the population traveling/gathering shows a larger impact on the peak prevalence and the total attack rate. When the initial R_0 _= 1.5 and 25% of the population starts traveling on Day 60 for 1 day, the peak prevalence increases from 2.73% (baseline) to 3.04% (approximately a 11% relative increase), significantly greater than the 4% relative peak prevalence increase (compared to baseline) when only 10% of the population travels on Day 60. Smaller mass gatherings (i.e., 1%-5% of the population) do not result in substantial increases in the peak prevalence and the total attack rate. Tables 2, 3, 4 show that this observation holds for other initial R_0 _values as well.

### Risk for Travelers' Families under the Non-Holiday Setting

To study the potential increase of the infection risk for the people traveling/gathering and for their family members (i.e., the impact of secondary transmissions), we compare the prevalence and the total attack rate in the non-Holiday setting to the baseline scenarios, for the population of travelers/gatherers and their family members.

When the initial R_0 _= 1.5 and 10% of the population is on travel during a 1-day traveling period beginning at Day 60 (or Day 30), the value of the peak prevalence is 2.97% (or 2.86%, respectively) and the total attack rate is 53.5% (or 53.0%, respectively) among the population of travelers/gatherers and their family members, while the peak prevalence in the entire population is 2.85% (or 2.78%, respectively) with a total attack rate 51.3% (or 51.0%, respectively). Please refer to [Additional file 1] for more details.

The peak prevalence value and the total attack rate for the persons who travel or attend mass gatherings and their family members are higher than the corresponding average values for the entire population when the traveling or mass gatherings occur before the epidemic peak. Even if the traveling period starts at Day 90 (20 days after the epidemic peak in the baseline scenario), the total attack rate for the travelers and their families is 53.0%, still higher than that for the entire state (51.0%).

### Regional Impact of Traveling and Mass Gatherings

**Figure 4:**
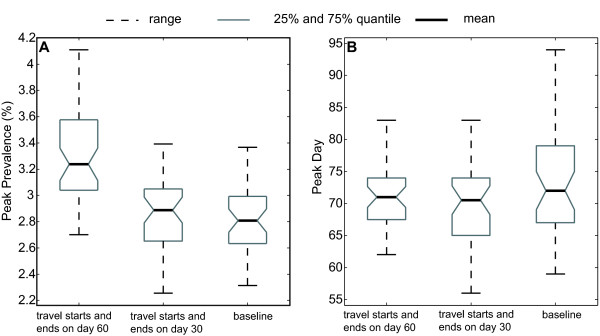
**Peak prevalence value and peak day in Bibb County**. The box plots show the range (with maximum and minimum, dotted line), 25% (lower gray line) and 75% (upper gray line) percentile, and the mean value (solid black line) for the peak prevalence (4A) and the peak day (4B) in Bibb County. Here 50% of the population from Bibb County and its nearest 5 counties travels and mixes with 9.5% of the population from other counties in the traveling group. The initial R_0 _= 1.5, the traveling period lasts for 1 day, and it starts on Day 30, 60, or no traveling (baseline).

The aforementioned scenarios assume that the proportion of persons traveling/gathering are uniform throughout the entire state; however, mass gatherings may disproportionately involve residents of certain areas or neighborhoods (e.g., residents closer to the mass gathering event may be more likely to attend than persons remote). Therefore an additional set of scenarios explores the impact of regional differences in traveling and mass gatherings under the non-Holiday setting. Figure 4 depicts the scenarios when the initial R_0 _= 1.5, the traveling period is 1 day, and 50% of the population in Bibb County and its nearest 5 counties [28] are mixing in the traveling group with 9.5% of the population traveling from all other counties (resulting in 10.4% total of the entire population on travel). Figure 4 shows the maximum and minimum, the 25% and 75% percentiles, and the mean of the peak prevalence value and the peak day for Bibb County (from 50 replications) with traveling starting on Day 30, Day 60, and without traveling (baseline scenario).

As shown in Figure 4(A), when the traveling/mass gathering starts on Day 60 and lasts for 1 day, the peak prevalence in Bibb County can reach as high as 4% in some experiments (compared to 2.82% in the entire state). The average peak prevalence is 3.32%, and the average total attack rate is 50.1%, which are higher than the baseline value of peak prevalence (2.82%) and total attack rate (48.9%) for Bibb County.

Moreover, Figure 4(B) indicates that the traveling/mass gatherings occurring before the peak prevalence day (e.g., Day 30) can synchronize the timing of the epidemic curves in a local county and in the entire state. In the baseline case, the day when the prevalence peaks in Bibb County can appear as late as Day 95, which is 25 days after the peak day in the entire state. With traveling/mass gathering occurring on Day 30, the peak day in Bibb County is mostly reached before Day 75 (with 75% chance); and furthermore, in some experiments, the peak day can occur as early as Day 56 due to the early introduction of seed infections to the local area.

## Discussion

Our simulation experiments identified situations where mass traveling or gatherings that occur shortly before the epidemic peak may worsen or alter the course of the influenza epidemic (e.g., resulting in a higher peak prevalence and total attack rate and in some cases generating two epidemic peaks), which may substantially affect planning and potentially strain healthcare facilities and resources. This impact can be greatest on the local communities hosting the mass gatherings. Therefore, public health officials, local authorities, and other decision makers may consider closely monitoring, postponing or cancelling public gatherings near the peak of an epidemic. Moreover, pandemic surveillance and other responses should not necessarily be slowed even after a large decline in influenza activity since a second epidemic peak may occur after Holiday traveling. Conversely, our experiments suggest that mass traveling or gatherings may have little effect when occurring relatively early or past the peak in an epidemic (with high enough herd immunity achieved [45-48]).

Our study emphasizes the impact of social mixing patterns and the creation and distribution of immune individuals on the progression of an epidemic. When individuals mix in households, schools, and workplaces without major changes, they can generate pockets of adequate herd immunity to prevent additional transmission. In other words, if a large percentage of individuals at one's workplace and household are immune then one's risk of infection may be low, even though many infectious individuals are still in the population. This is because individuals tend to stick with their typical social contacts and do not mix with a majority of the population. However, a mass gathering brings together people that normally would not mix, i.e., it brings together susceptible and infectious individuals that would not have interacted otherwise, thus potentially worsening the epidemic.

Additionally, the differences between the durations of the mass gathering and the pathogen's infectious period can substantially alter the impact of mass gathering. When the mass gathering period is shorter than the pathogen's infectious period (e.g., when the mass gathering is 1-2 days versus 3-4 days for the infectious period), mass gathering creates new infectious individuals who then return to their households, workplaces, and schools to infect their standard social networks, thereby worsening the epidemic. Conversely, when the mass gathering lasts as long as or longer than the pathogen's infectious period (e.g., when the mass gathering infectious period is 3-4 days and the pathogen's infectious period is 3-4 days), mass gathering can actually act as a mass immunization or mass quarantine event, keeping people in one location while they are infectious and then returning them to their social networks only after they are immune. Sensitivity analyses that increase the average infectious period to 7-8 days [41,42] support this conclusion.

Previous studies have suggested that social mixing patterns play an important role in influenza spread, and social distancing measures such as school closure may be able to mitigate an epidemic [9-12,17-26,29,49]. But few studies have focused on the opposite of social distancing, i.e., social gatherings, during an epidemic. There have been studies on the potential effects of national and international travel restrictions, e.g., border closures or international air travel restrictions, but less on local or regional travel [11]. Our study demonstrates how social mixing dynamics can be captured in a heterogeneous population, and it shows the impact on prevalence, peak timing, and secondary transmissions within families or regions.

Certainly, cancelling or postponing mass gatherings near the epidemic peak can be challenging. As seen during the 2009 H1N1 pandemic, it can be difficult to determine the current and anticipated future status of an ongoing epidemic. Moreover, changing a previously scheduled event can have economic and logistic consequences. In some cases, the scheduled date of a mass gathering can have significance. For example, Memish et al. [50] discussed the global religious event Hajj (pilgrimage by Muslims to Saudi Arabia, attracting more than 2.5 million pilgrims from the whole world every year), which is difficult to cancel during a pandemic.

The alternative to changing the scheduling of an event is close monitoring and enforcement of hygienic measures and precautions during the event. Memish et al. [50] and Rashid et al. [51] presented several recommendations for local governments to follow, including screening, surveillance, and most importantly, encouraging attendees from high risk groups (e.g., elderly and pregnant women) to postpone their participation in the event. Also, reducing the length and the scale of an event could be less drastic ways of reducing disease transmission. Even if an event cannot be cancelled, knowing that it may increase the overall attack rate and peak prevalence could help public health decision makers prepare (e.g., increasing health care resource availability and surge capacity).

### Limitations

Computer simulations by definition are simplifications of real life. Rather than make decisions, they can identify potentially important factors and relationships for decision makers. Our model does incorporate a number of assumptions and cannot fully capture every possible factor or effect. For example, we assume homogeneous mixing within the traveling/mass gathering group during the traveling/mass gathering period. In real life, people may not have contact with every attendee in a mass gathering. Also, mass gathering events are not equivalent. Some may involve closer and more extended contact than others. The type of venue and location can play a significant role. Different events can involve people of different ages, socioeconomic status, and potentially health status. Although we conducted a wide-range of sensitivity analyses, it is not possible to explore every possible combination of parameters.

## Conclusions

When they occur close to the peak of an epidemic, mass gatherings and traveling could worsen the overall attack rate and the peak prevalence. However, such changes in social mixing may have little effect when they occur earlier or later in the course of an epidemic. Public health decision makers may use this information to help decide whether to postpone, cancel, monitor, or enforce infection control measures during a mass gathering or Holiday season.

## Competing interests

The authors declare that they have no competing interests.

## Authors' contributions

PS participated in the design of the study, implemented the computational model and data analysis, and drafted the manuscript. PK and JLS carried out the study design and coordination and participated in data analysis. BYL served as advisor on disease transmission details and participated in the study's design and coordination. All authors contributed to interpretation of findings, preparing the manuscript, read and approved the final manuscript.

## Pre-publication history

The pre-publication history for this paper can be accessed here:

http://www.biomedcentral.com/1471-2458/10/778/prepub

## Supplementary Material

Additional file 1 **Model description and calibration**. The additional file contains the description of the agent-based simulation model and the detailed calibration for the model. It also contains the calculation of the prevalence value for the travelers and their family members in the non-Holiday setting. Click here for file
