# Supplementary material for: The impact of mass gatherings and holiday traveling on the course of an influenza pandemic: a computational model
Source: BMC Public Health. 2010 Dec 21;10:778. doi: 10.1186/1471-2458-10-778 (PMC3022852; doi:10.1186/1471-2458-10-778)
Supplement: Additional file 1 — Model description and calibration. The additional file contains the description of the agent-based simulation model and the detailed calibration for the model. It also contains the calculation of the prevalence value for the travelers and their family members in the non-Holiday setting. [file 1471-2458-10-778-S1.PDF]

Technical Appendix for "The Impact of Mass Gatherings and Holiday Traveling on the Course of an Influenza Pandemic: A Computational Model"

Pengyi Shi, Pinar Keskinocak, Julie L Swann, Bruce Y Lee

December 18, 2010

1 Disease Spread Model

The disease spread model consists of two parts: (i) the natural disease progression within an infected individual, and (ii) the contact network of each individual in the population.

1.1 Natural Disease Progression

We use a detailed SEIR (Susceptible–Exposed–Infected–Recovered) model for part (i) [1]. Each infected individual progresses from S (susceptible) through E (exposed but not yet infectious) to status I_P (presymptomatic). Then the individual has probability p_A of not developing symptoms and becoming I_A (infectious and asymptomatic), or becoming I_S (infectious and symptomatic) with probability ($1–p_A$). With probability p_H a symptomatic individual is hospitalized (H) and with probability p_D a hospitalized individual dies (D). R is the state of recovered, and a recovered individual is immune. This natural disease progression for influenza is depicted in Figure 1 of [1]. We list the values of part of the parameters here in Table S1 for reader's convenience (also refer to Table 1 in the main text).

1.2 Contact Network

We used a group model to construct the contact network in part (ii) (see Fig. 1 in the main text for an example). There are three levels of mixing for each individual during the "regular" period (household, peer group, and community), and four potential levels of mixing during the "travel/mass gathering" period (household, peer group, community, and the "traveling/mass gathering" group). Here the peer group refers to classrooms for children or workplaces for working adults, and the traveling group models temporary mass gathering locations, e.g., airports, shopping malls, convention centers, attractions, festivals, etc. A susceptible individual can become infected through contacts with his/her family members, classmates/colleagues, or random contacts with someone when going to community places such as grocery stores, or when travelling/attending mass gathering events.

The contact network model is age-structured as we divided the population into five age groups: 0-5, 6-11, 12-18, 19-64, $\geq$65 years [6, 9]. The first three groups represent children, who are assumed to have higher susceptibility and infectivity compared to adults. The average classroom sizes are 14, 20, and 30 for the three children's groups, respectively [8]. Individuals in the fourth group are working adults. The workplace size

Table S1. Values of the Key Parameters.

**Parameter**	**Explanation**	**Base Value**	**Reference**
p_A	Probability of infected individual remaining asymptomatic throughout course of infection	0.4 for working adults, 0.25 for others	[1, 2, 4, 5]
p_H	Probability of symptomatic individual requiring hospitalization	0.18 for age 0-5, 0.12 for 65+ and 0.06 for others	[1, 5]
p_D	Probability hospitalized individual not surviving	0.344 for age 0-5, 0.172 for others	[1, 6]
Duration of $E + I_P$	Duration of exposed and presymptomatic stage	Weibull with mean 1.48 and std. dev. 0.47, and offset 0.5	[1, 7]
Duration of I_S	Duration of symptomatic stage	Exponential with mean 2.7313 (mean = 7 in the sensitivity analysis)	[1]
Duration of I_A	Duration of asymptomatic stage	Exponential with mean 1.63878 (mean = 7 in the sensitivity analysis)	[1]
Duration of I_H	Duration of hospitalized stage	Exponential with mean 14	[1, 7]
Initial R_0	Reproductive rate (average number of secondary cases generated by each infected individual) before social mixing changes introduced	1.3, 1.5, and 1.8	[1, 3, 5, 7, 11]
θ	Proportion of transmission that occurs at presymptomatic/asymptomatic stage	0.3	[1]
ω	Proportion of infections generated by individuals who are asymptomatic	0.15	[1]
γ	Proportion of transmission that occurs outside the households	0.7	[3]
δ	Proportion of transmission outside the home that occurs in the community	0.5	[3]
m_i	Relative infectivity of the ith person	1.3158 for children, 0.8772 for adults	[6, 9]
S_i	Relative susceptibility of the ith person	1.1036 for children, 0.9597 for adults, 0 if not susceptible	[6, 9]

for working adults is a Poisson random variable with mean 20 (maximum 1000). The last group constitutes the elderly, and they do not mix in peer groups during the daytime. We list the distribution and the size of the social groups in Table 1 in the main text.

To calibrate the disease spread through the contact network, the following parameters need to be estimated: the coefficient of transmission β, relative hazards of an infected individual at presymptomatic and asymptomatic stages (h_{PS} and h_{AS}) to symptomatic stage, and relative hazards in peer groups, community, and traveling/mass gathering group (if any) to households (h_{PG}, h_C and h_T). The size of the social group a person belongs to determines how many persons he/she can "contact" with (known as "group model" [14]). The relative hazard rates (h_{PG}, h_C and h_T) adjust the probability of infection between two individuals' contacts in different social groups (the probability is the highest when contacts occurring in households, medium when occurring in the peer groups, lowest when occurring in the community locations or in the traveling group). These parameters are used to define different disease settings and to calculate the "force of infection"[1], which determines the chance of a susceptible person getting infected and is crucial in the discrete-event simulation.

We used a similar nonlinear technique as in [1] to calculate the parameters, but we adjusted the calculation for our age-based model as well as for the the traveling/mass gathering group. The calculation of these parameters and the corresponding "force of infection" are as follows.

1.2.1 Estimate of Parameters for Regular Period

During a regular period (i.e., no traveling or mass gathering occurs), there are three levels of mixing for each individual: household, peer group, and community. Thus we want to estimate β, h_{PS}, h_{AS}, h_{PG}, and h_C here. Let r_{XY} be the average number of people infected in Y by an individual who is at stage X where Y is the household (H), peer group (PG) or the community (C) and X is the presymptomatic (P), asymptomatic (A) or symptomatic (S) stage. The r_{XY} values are calculated as follows:

$$
\begin{aligned}
r_{PH} &= \sum_{n=1}^{7} p_n(n-1)(1-\phi_P(\frac{h_{PS}\beta}{2n})) \\
r_{AH} &= p_A \sum_{n=1}^{7} p_n(n-1)\phi_P(\frac{h_{PS}\beta}{2n})(1-\phi_A(\frac{h_{AS}\beta}{2n})) \\
r_{SH} &= (1-p_A)\sum_{n=1}^{7} p_n(n-1)\phi_P(\frac{h_{PS}\beta}{2n})(1-\phi_S(\frac{\beta}{2n})) \\
r_{P,PG} &= (q_1n_1+q_2n_2+q_3n_3+q_4n_4+q_5n_5)(1-\phi_P(\frac{h_{PS}h_{PG}\beta}{2})) \\
r_{A,PG} &= p_A(q_1n_1+q_2n_2+q_3n_3+q_4n_4+q_5n_5)\phi_P(\frac{h_{PS}h_{PG}\beta}{2})(1-\phi_A(\frac{h_{AS}h_{PG}\beta}{2})) \\
r_{S,PG} &= (1-p_A)\cdot[(q_1n_1+q_2n_2+q_3n_3)\phi_P(\frac{h_{PS}h_{PG}\beta}{2})(1-\phi_S(0))+(q_4n_4+q_5n_5)\phi_P(\frac{h_{PS}h_{PG}\beta}{2})(1-\phi_S(\frac{h_{PG}\beta}{4})) \\
r_{PC} &= N(1-\phi_P(\frac{h_{PS}h_C\beta}{2N})) \\
r_{AC} &= p_AN\phi_P(\frac{h_{PS}h_C\beta}{2N})(1-\phi_A(\frac{h_{AS}h_C\beta}{2N})) \\
r_{SC} &= (1-p_A)N\phi_P(\frac{h_{PS}h_C\beta}{2N})(1-\phi_S(\frac{h_C\beta}{2N}))
\end{aligned}
$$

where q_i is the proportion of population in age group i for $i = 1, \cdots, 5$, p_A is the probability that a presymptomatic individual does not develop symptoms, and n_i is the average size of peer groups for age group i. In our model, the maximum household size is 7, p_n is the probability that an individual lives in a household size of n, and N is the total number of people in the considered area [10]. $\phi_X(h) = E(e^{-hD_X})$ is the probability that an infection does not occur between two individuals during phase X ($X \in \{P, A, S\}$, D_X is the duration of stage X) for a constant hazard of infection h.

The r_{XY} values can also be represented by the following given disease parameters: the initial R_0; θ, the proportion of transmission that occurs at either presymptomatic or asymptomatic stage; ω, the proportion of infections generated by individuals who are never symptomatic; γ, the proportion of transmission that occurs outside the households; and δ, the proportion of transmission outside the home that occurs in the community. The values of $R_{0,}$ $\theta,$ $\omega,$ $\gamma,$ and δ are given in Table S1 (aslo see Table 1 in the main text).

$$
\begin{aligned}
R_0 &= r_{PH}+r_{AH}+r_{SH}+r_{P,PG}+r_{A,PG}+r_{S,PG}+r_{PC}+r_{AC}+r_{SC} \\
\theta &= \frac{r_{PH}+r_{AH}+r_{P,PG}+r_{A,PG}+r_{PC}+r_{AC}}{R_0} \\
\omega &= \frac{r_{AH}+p_Ar_{PH}+r_{A,PG}+p_Ar_{P,PG}+r_{AC}+p_Ar_{PC}}{R_0} \\
\gamma &= \frac{r_{P,PG}+r_{A,PG}+r_{S,PG}+r_{PC}+r_{AC}+r_{SC}}{R_0} \\
\delta &= \frac{r_{PC}+r_{AC}+r_{SC}}{r_{P,PG}+r_{A,PG}+r_{S,PG}+r_{PC}+r_{AC}+r_{SC}}
\end{aligned}
$$

When we relate the r_{XY} values with the initial R_0 (i.e., the first equation), we use the idea that the average number of secondary cases from a "typical" infectious individual generated in his/her social groups is equal to R_0. Similarly we derive the other four equations. Based on R_0, θ, ω, γ, and δ, we can solve the above nonlinear equations and obtain the values for β, h_{PS}, h_{AS}, h_{PG}, and h_C.

In the disease spread model, we use the discrete-event simulation, i.e., we simulate the time of the next infection and choose the person that will be infected. The next infection time is generated by calculating the instantaneous "force of infection" for each individual [1]. The instantaneous "force of infection" represents the rate at which susceptible persons become infected, and is determined by the number of infectious persons (i.e., persons in the presymptomatic, asymptomatic and symptomatic stages) at the current time as well as the parameters β, h_{PS}, h_{AS}, h_{PG}, and h_C. The force of infection experienced by the ith person during the day (λ_i^D) and during the night (λ_i^N) in the regular period are calculated as follows:

$$\begin{aligned}
\lambda_i^D &= S_i \sum_{j=1}^{N} (\delta_{ij}^{PG} m_j \epsilon_j h_{PG} h_{X,j} \beta + \delta_{ij}^{C} \frac{m_j h_C h_{X,j} \beta}{N_i}) \\
\lambda_i^N &= S_i \sum_{j=1}^{N} (\delta_{ij}^{H} \frac{m_j h_{X,j} \beta}{n_i^{HA}} + \delta_{ij}^{C} \frac{m_j h_C h_{X,j} \beta}{N_i})
\end{aligned}$$

where S_i and m_i are the relative susceptibility and infectivity of the ith person (see Table S1). N_i is the number of population in the ith person's community and n_i^{HA} is the active household size of this person where dead and hospitalized persons are not counted. δ_{ij}^Y ($Y \in \{H,\ PG,\ C\}$) is the indicator functions defined for location Y (households, peer groups or community)

$$\delta_{ij}^Y = \begin{cases} 1 & \text{if person i and j are in the same location } Y \\ 0 & \text{otherwise} \end{cases}$$

and ϵ_j is the indicator showing whether person j withdraws from workplace or school:

$$\epsilon_j = \begin{cases} 1 & \text{if person j mixes in the peergroup} \\ 0 & \text{if person j withdraws from the peergroup} \end{cases}$$

We assume that 100% symptomatic children and 50% symptomatic adults withdraw from their peergroups [1]. Finally, $h_{X,j}$ is the relative hazard rate of the jth person if he/she is in the disease stage X, i.e.,

$$h_{X,j} = \begin{cases} h_{PS} & \text{if person j is in the presymptomatic stage} \\ h_{AS} & \text{if person j is in the asymptomatic stage} \\ 1 & \text{if person j is in the symptomatic stage} \\ 0 & \text{otherwise} \end{cases}$$

After the infected individual is selected, the disease progresses according to what we described in section 1.1.

1.2.2 Estimate of Parameters for Traveling Period: Non-Holiday Setting

During the traveling period in the non-Holiday scenario, we have different mixing patterns for the persons who are travelling or attending mass gatherings, and those are not:

1. Person not travelling/attending mass gatherings: retains his/her usual mixing pattern in the regular period, i.e. mix in household (night), peergroup (day) and community (day/night)

2. Person travelling/attending mass gatherings: only mix in the traveling/mass gathering group (day/night)

So the infection hazard rate (h_{PS}, h_{AS}, h_{PG}, h_C) and the transmission rate β remain the same as in the regular period (see section 1.2.1) for those persons who are not on travel or attending mass gatherings. However, for the persons who travel or attend mass gathering events, we need to calculate their specific infection hazard rate ($\tilde{h}_{PS}$ and $\tilde{h}_{AS}$) and the transmission rate $\tilde{\beta}$ within the traveling/mass gathering group.

Let r_{XT} be the average number of people infected in T (traveling/mass gathering group) by an individual who is at stage X (X can be the presymptomatic (P), asymptomatic (A) or symptomatic (S) stage):

$$\begin{aligned}
r_{PT} &= N_T(1 - \phi_P(\frac{\tilde{h}_{PS}\tilde{\beta}}{N_T})) \\
r_{AT} &= p_A N_T \phi_P(\frac{\tilde{h}_{PS}\tilde{\beta}}{N_T})(1 - \phi_A(\frac{\tilde{h}_{AS}\tilde{\beta}}{N_T})) \\
r_{ST} &= (1 - p_A) N_T \phi_P(\frac{\tilde{h}_{PS}\tilde{\beta}}{N_T})(1 - \phi_S(\frac{\tilde{\beta}}{N_T}))
\end{aligned}$$

where N_T is the number of population on travel. We then use R_0, θ, and ω with the same value described in section 1.2.1 to determine r_{XT} and then further calculate $\tilde{\beta}$, $\tilde{h}_{PS}$ and $\tilde{h}_{AS}$:

$$\begin{aligned}
R_0 &= r_{PT} + r_{AT} + r_{ST} \\
\theta &= \frac{r_{PT} + r_{AT}}{R_0} \\
\omega &= \frac{r_{AT} + p_A r_{PT}}{R_0}.
\end{aligned}$$

For a susceptible person who travels or attends mass gathering events, if he/she becomes infected during the traveling period, the source of infection is from contacting with other people in the traveling group (100% chance); while those susceptible persons who are not traveling or attending mass gatherings may become infected from mixing in the households, in the peergroups, or in the communities with 30%, 35%, and 35% chance, respectively (see Table S1).

Therefore, if we assume that 10% of the population is on travel/attending mass gatherings, a susceptible person can become infected from mixing in the household, in the peergroup, in the communities, or in the traveling group with 27%, 31.5%, 31.5%, and 10% chance, respectively during the traveling period. During the regular period, a susceptible person can become infected from mixing in the households, in the peergroups, or in the communities with 30%, 35%, and 35% chance, respectively.

The force of infection experienced by the ith person during the day (λ_i^D) and during the night (λ_i^N) in the traveling period (**non-Holiday setting**) are calculated as follows:

$$\lambda_i^D = S_i \cdot \left[(1-\sigma_i)\cdot\sum_{j=1}^{N}(\delta_{ij}^{PG} m_j \epsilon_j h_{PG} h_{X,j}\beta + \delta_{ij}^{C}\frac{(1-\sigma_j)m_j h_C h_{X,j}\beta}{N_i^A}) + \sigma_i \cdot \sum_{j=1}^{N}\delta_{ij}^{T}\frac{\sigma_j m_j \tilde{h}_{X,j}\tilde{\beta}}{N_T}\right]$$

$$\lambda_i^N = S_i \cdot \left[(1-\sigma_i)\cdot\sum_{j=1}^{N}(\delta_{ij}^{H}\cdot\frac{(1-\sigma_j)m_j h_{X,j}\beta}{n_i^{HA}} + \delta_{ij}^{C}\cdot\frac{(1-\sigma_j)m_j h_C h_{X,j}\beta}{N_i^A}) + \sigma_i \cdot \sum_{j=1}^{N}\delta_{ij}^{T}\frac{\sigma_j m_j \tilde{h}_{X,j}\tilde{\beta}}{N_T}\right]$$

In the non-Holiday setting, during the day, the next infection event (determined by λ_i^D) can occur in the peergroup, in the traveling group, or in the community; while the next infection event during the night (determined by λ_i^N) occurs in the household, in the traveling group, or in the community.

Here S_i and m_i are the relative susceptibility and infectivity of the ith person (see Table S1). N_i^A is the number of population in the ith person's community except those persons who are traveling or attending mass gatherings, and n_i^{HA} is the active household size of this person where persons who are dead, hospitalized, or traveling are not counted. N_T is the number of population in the traveling group, i.e., the number of persons who are traveling or attending mass gatherings.

δ_{ij}^Y ($Y \in \{H,\ PG,\ C,\ T\}$) is the indicator functions defined for location Y (households, peer groups, community, or the traveling group)

$$\delta_{ij}^Y = \begin{cases} 1 & \text{if person i and j are in the same location } Y \\ 0 & \text{otherwise} \end{cases}$$

and ϵ_j is the indicator showing whether person j withdraws from workplace or school:

$$\epsilon_j = \begin{cases} 1 & \text{if person j mixes in the peergroup} \\ 0 & \text{if person j withdraws from the peergroup} \end{cases}$$

We still assume that 100% symptomatic children and 50% symptomatic adults withdraw from their peergroups; besides, the persons who are traveling or attending mass gathering events also withdraw from their peergroups. The indicator variable σ_i is to show whether person i is traveling or attending mass gatherings:

$$\sigma_i = \begin{cases} 1 & \text{if person i is traveling or attending mass gathering events} \\ 0 & \text{otherwise} \end{cases}$$

Finally, $h_{X,j}$ is the relative hazard rate of the jth person if he/she is in the disease stage X and not traveling, and $\tilde{h}_{X,j}$ is the relative hazard rate if this person is traveling:

$$\tilde{h}_{X,j} = \begin{cases} \tilde{h}_{PS} & \text{if person j is in the presymptomatic stage} \\ \tilde{h}_{AS} & \text{if person j is in the asymptomatic stage} \\ 1 & \text{if person j is in the symptomatic stage} \\ 0 & \text{otherwise} \end{cases}$$

After the infected individual is selected, the disease progresses according to what we described in section 1.1.

1.2.3 Estimate of Parameters for Traveling Period: Holiday Setting

During the traveling period with the Holiday settings, we have three different mixing patterns in total, one for the persons on travel and two for those who are not:

1. Working adults who are not on travel and still go to work during the day time: keep his/her usual mixing patterns, i.e., mix in household (night), peergroup (day) and community (day/night)
2. Children, elderly, and working adults who are not on travel but stay at home all day: mix in houshold (day/night) and community (day/night)
3. Persons on travel with their family members during Holiday: mix in traveling/mass gathering group (day) and household (day/night).

So the infection hazard rate (h_{PS}, h_{AS}, h_{PG}, h_C) and the transmission rate β remain the same as in the regular period (see section 1.2.1) for the working adults who are not on travel and still go to work. However, for the person on travel and the persons stay at home all day, we need to calculate their new infection hazard rates and transmission rate.

Persons not traveling and staying at home all day

Let $\tilde{r}_{XY}$ be the average number of people infected in Y by an individual who is at stage X (i.e., X can be the presymptomatic (P), asymptomatic (A) or symptomatic (S) stage; Y can be the household (H) or the community (C)):

$$
\begin{aligned}
\tilde{r}_{PH} &= \sum_{n=1}^{7} p_n(n-1)(1-\phi_P(\frac{\tilde{h}_{PS}\tilde{\beta}}{n})) \\
\tilde{r}_{AH} &= p_A\sum_{n=1}^{7} p_n(n-1)\phi_P(\frac{\tilde{h}_{PS}\tilde{\beta}}{n})(1-\phi_A(\frac{\tilde{h}_{AS}\tilde{\beta}}{n})) \\
\tilde{r}_{SH} &= (1-p_A)\sum_{n=1}^{7} p_n(n-1)\phi_P(\frac{\tilde{h}_{PS}\tilde{\beta}}{n})(1-\phi_S(\frac{\tilde{\beta}}{n})) \\
\tilde{r}_{PC} &= N(1-\phi_P(\frac{\tilde{h}_{PS}\tilde{h}_C\tilde{\beta}}{N})) \\
\tilde{r}_{AC} &= p_A N\phi_P(\frac{\tilde{h}_{PS}\tilde{h}_C\tilde{\beta}}{N})(1-\phi_A(\frac{\tilde{h}_{AS}\tilde{h}_C\tilde{\beta}}{N})) \\
\tilde{r}_{SC} &= (1-p_A)N\phi_P(\frac{\tilde{h}_{PS}\tilde{h}_C\tilde{\beta}}{N})(1-\phi_S(\frac{\tilde{h}_C\tilde{\beta}}{N}))
\end{aligned}
$$

We then use R_0, θ, ω and γ (the values are shown in Table S1) to determine $\tilde{r}_{XT}$ and further calculate$\tilde{\beta}$, $\tilde{h}_{PS}$, $\tilde{h}_{AS}$, and $\tilde{h}_C$:

$$\begin{aligned}
R_0 &= \tilde{r}_{PH} + \tilde{r}_{AH} + \tilde{r}_{SH} + \tilde{r}_{PC} + \tilde{r}_{AC} + \tilde{r}_{SC} \\
\theta &= \frac{\tilde{r}_{PH} + \tilde{r}_{AH} + \tilde{r}_{PC} + \tilde{r}_{AC}}{R_0} \\
\omega &= \frac{\tilde{r}_{AH} + p_A\tilde{r}_{PH} + \tilde{r}_{AC} + p_A\tilde{r}_{PC}}{R_0} \\
\gamma &= \frac{\tilde{r}_{PC} + \tilde{r}_{AC} + \tilde{r}_{SC}}{R_0}.
\end{aligned}$$

Persons traveling or attending mass gatherings

Let $\bar{r}_{XY}$ be the average number of people infected in Y by an individual who is at stage X (i.e., X can be the presymptomatic (P), asymptomatic (A) or symptomatic (S) stage; Y can be the household (H) or the traveling group (T)):

$$\begin{aligned}
\bar{r}_{PH} &= \sum_{n=1}^{7} p_n(n-1)(1-\phi_P(\frac{\bar{h}_{PS}\bar{\beta}}{n})) \\
\bar{r}_{AH} &= p_A \sum_{n=1}^{7} p_n(n-1)\phi_P(\frac{\bar{h}_{PS}\bar{\beta}}{n})(1-\phi_A(\frac{\bar{h}_{AS}\bar{\beta}}{n})) \\
\bar{r}_{SH} &= (1-p_A) \sum_{n=1}^{7} p_n(n-1)\phi_P(\frac{\bar{h}_{PS}\bar{\beta}}{n})(1-\phi_S(\frac{\bar{\beta}}{n})) \\
\bar{r}_{PT} &= N_T(1-\phi_P(\frac{\bar{h}_{PS}\bar{h}_T\bar{\beta}}{2N_T})) \\
\bar{r}_{AT} &= p_A N_T \phi_P(\frac{\bar{h}_{PS}\bar{h}_T\bar{\beta}}{2N_T})(1-\phi_A(\frac{\bar{h}_{AS}\bar{h}_T\bar{\beta}}{2N_T})) \\
\bar{r}_{ST} &= (1-p_A) N_T \phi_P(\frac{\bar{h}_{PS}\bar{h}_T\bar{\beta}}{2N_T})(1-\phi_S(\frac{\bar{h}_T\bar{\beta}}{2N_T}))
\end{aligned}$$

where N_T is the number of population on travel. We then use R_0, θ, ω (the values are shown in Table S1) and $\tilde{\gamma}$ (=0.4, the proportion of transmission that occurs in the traveling group) to determine $\bar{r}_{XY}$ and then further calculate$\bar{\beta}$, $\bar{h}_{PS}$, $\bar{h}_{AS}$, and $\bar{h}_T$:

$$\begin{aligned}
R_0 &= \bar{r}_{PH} + \bar{r}_{AH} + \bar{r}_{SH} + \bar{r}_{PT} + \bar{r}_{AT} + \bar{r}_{ST} \\
\theta &= \frac{\bar{r}_{PH} + \bar{r}_{AH} + \bar{r}_{PT} + \bar{r}_{AT}}{R_0} \\
\omega &= \frac{\bar{r}_{AH} + p_A\bar{r}_{PH} + \bar{r}_{AT} + p_A\bar{r}_{PT}}{R_0} \\
\gamma &= \frac{\bar{r}_{PT} + \bar{r}_{AT} + \bar{r}_{ST}}{R_0}.
\end{aligned}$$

The force of infection experienced by the ith person during the day (λ_i^D) and during the night (λ_i^N) in the traveling period (**Holiday setting**) are calculated as follows:

$$\lambda_i^D = \begin{cases} S_i \cdot \sum_{j=1}^{N}(\delta_{ij}^{PG} m_j \epsilon_i \epsilon_j h_{PG} h_{X,j}\beta + \delta_{ij}^{C}\frac{(1-\sigma_j)m_j h_C h_{X,j}\beta}{N_i^A}) & \text{if person i retains mixing patterns } (\sigma_i = 0,\ \epsilon_i = 1) \\ S_i \cdot \sum_{j=1}^{N}(\delta_{ij}^{H} \cdot \frac{(1-\epsilon_j)m_j \tilde{h}_{X,j}\tilde{\beta}}{n_i^{HA}} + \delta_{ij}^{C}\frac{(1-\sigma_j)m_j \tilde{h}_C \tilde{h}_{X,j}\tilde{\beta}}{N_i^A}) & \text{if person i stays at home all day } (\sigma_i = 0,\ \epsilon_i = 0) \\ S_i \cdot \sum_{j=1}^{N}(\delta_{ij}^{H} \cdot \frac{m_j \bar{h}_{X,j}\bar{\beta}}{n_i^{HA}} + \delta_{ij}^{T}\frac{\sigma_j m_j \bar{h}_T \bar{h}_{X,j}\bar{\beta}}{N_T}) & \text{if person i is traveling } (\sigma_i = 1,\ \epsilon_i = 0) \end{cases}$$

$$\lambda_i^N = \begin{cases} S_i \cdot \sum_{j=1}^{N} \delta_{ij}^{H} \cdot \frac{m_j h_{X,j}\beta}{n_i^{HA}} & \text{if person i is not traveling } (\sigma_i = 0) \\ S_i \cdot \sum_{j=1}^{N} \delta_{ij}^{H} \cdot \frac{m_j \bar{h}_{X,j}\bar{\beta}}{n_i^{HA}} & \text{if person i is traveling } (\sigma_i = 1) \end{cases}$$

In the Holiday setting, the next infection event during the night (determined by λ_i^N) only occur in the household and in the community, while during the day, the next infection event (determined by λ_i^D) can occur in the household, in the peergroup, in the traveling group, or in the community.

Here S_i and m_i are the relative susceptibility and infectivity of the ith person (see Table S1). N_i^A is the number of population in the ith person's community except those persons who are traveling or attending mass gatherings, and n_i^{HA} is the active household size of this person where dead and hospitalized persons are not counted. N_T is the number of population in the traveling group, i.e., the number of persons who are traveling or attending mass gatherings.

δ_{ij}^Y ($Y \in \{H,\ PG,\ C,\ T\}$) is the indicator functions defined for location Y (households, peer groups, community, or the traveling group)

$$\delta_{ij}^Y = \begin{cases} 1 & \text{if person i and j are in the same location } Y \\ 0 & \text{otherwise} \end{cases}$$

and ϵ_j is the indicator showing whether person j withdraws from workplace or school.

$$\epsilon_j = \begin{cases} 1 & \text{if person j mixes in the peergroup} \\ 0 & \text{if person j withdraw from the peergroup} \end{cases}$$

We still assume that 100% children and 50% adults withdraw from their peergroups during the Holiday season; besides, the persons who are traveling also withdraw from their peergroups. The indicator variable σ_i is to show whether person i is traveling or attending mass gatherings:

$$\sigma_i = \begin{cases} 1 & \text{if person i is traveling during Holiday} \\ 0 & \text{otherwise} \end{cases}$$

Finally, $h_{X,j}$ is the relative hazard rate of the jth person if he/she is in the disease stage X and does not change the mixing patterns; $\tilde{h}_{X,j}$ is the relative hazard rate if this person is not traveling and stay at home during the day; and $\bar{h}_{X,j}$ is the relative hazard rate for those traveling persons:

Table S2. Adjusted Paramters to Achieve the Attack Rates in the 1957 Pandemic.

**Parameter**	**Base Value**	**Adjusted Value**
p_A	0.4 for working adults, 0.25 for others	0.33 for age 0-18, 0.50 for age 19-64, 0.68 for age 65+
Initial R_0	1.3, 1.5, and 1.8	1.53
S_i	1.1036 for children, 0.9597 for adults, 0 if not susceptible	1.4236 for children, 0.8374 for adults, 0 if not susceptible

$$\bar{h}_{X,j} = \begin{cases} \bar{h}_{PS} & \text{if person j is in the presymptomatic stage} \\ \bar{h}_{AS} & \text{if person j is in the asymptomatic stage} \\ 1 & \text{if person j is in the symptomatic stage} \\ 0 & \text{otherwise} \end{cases}$$

After the infected individual is selected, the disease progresses according to what we described in section 1.1.

1.3 Model Validation

The current model is calibrated to match the attack rate in the 1918 pandemic (i.e., when the initial R_0=1.8, the clinical attack rate is 50% [1, 7]). We also did experiments to calibrate our model for other pandemics in the history, e.g., the initial R_0 value is tuned to be 1.53 to match the age-specific attack rates in the 1957 pandemic as shown in [12]. The adjusted R_0 value is consistent with the estimates 1.5-1.7 for the 1957 pandemic [3]. This provides us a way to validate the simulation model (similar way of validation has been used in other papers [5, 7, 13]).

Table S2 shows the values of adjusted parameters to achieve the age-specific clinical attack rates (proportion of symptomatic cases) in the 1957 pandemic. Table S3 reports the age-specific attack rates from [12] and from our simulation, respectively.

In summary, our disease spread model is based on several other simulation models used for influenza pandemic in the literature [1, 3, 4, 5, 7, 9]. However, most of the agent-based simulation models assume the social mixing patterns are stationary, which do not fit in our settings. That is the reason why we develop the new model and consider different mixing patterns during different periods. To calibrate the transmission for the new model, we calculate the transmission parameters separately for each person during the regular period and during the traveling period, which is not presented in other simulation models. Treating the people who change their mixing patterns differently from those retaining their mixing patterns provides more flexbility in modeling, and may be generalized to model other mixing scenarios besides holiday traveling and mass gathering events (e.g., patients seeking medical care and mix in the hospitals).

Table S3. Age-specific Attack Rates.

Age Group	Clinical Attack Rate in the 1957 Pandemic [12]	Clinical Attack Rate (simulation)
Age Group 1 (0-5 years)	32.17%	33.05%
Age Group 2 (6-11 years)	35.02%	35.37%
Age Group 3 (12-18 years)	38.44%	38.67%
Age Group 4 (19-64 years)	22.24%	21.88%
Age Group 5 ($\geq$65 years)	10.00%	10.04%
Total	24.72%	24.53%

Table S4. Proportion of the Population in Each Age Group.

Proportion of Each Age Group	Entire Population	Target Population
Age Group 1 (0-5 years)	8.62%	10.72%
Age Group 2 (6-11 years)	9.09%	11.26%
Age Group 3 (12-18 years)	9.98%	12.38%
Age Group 4 (19-64 years)	62.44%	56.93%
Age Group 5 ($\geq$65 years)	9.84%	8.68%

2 Calculating the Prevalence Level among the Travelers' Families

In the non-Holiday scenarios, we study the potential increase in the risk of getting infected for the persons who are on travel/attending mass gathering events as well as their family members. To do so, we keep track of the number of infections (i.e. number of symptomatic and asymptomatic persons) in a target population. A person is selected in this target pool if he/she is mixing in the "traveling/gathering group" during the traveling period, or belongs to the same household of a "traveler" or "attendee" of mass gatherings.

We want to compare the prevalence level (e.g., peak prevalence value, total attack rate) among the target population with the prevalence level in the entire state. However, the above method of selection results in different distribution of age groups in the target population from the distribution of age groups in the entire population, even though the "travelers/attendees" are sampled uniformly from the entire population. Table S4 shows that the target pool (persons on travel/attending mass gatherings and their family members) has a higher proportion of children, i.e. a higher proportion of persons from the first three age groups, than that in the entire population.

Note that in the simulation model, children may not necessarily travel with their family members in the non-Holiday scenarios (e.g., they may travel with neighbors or relatives to watch football games), but we do assume that children cannot live in households by themselves. Those persons living in the single household are either workding adults or elderly (the proportion of single household is around 10% [10]). Therefore, when we select the travelers/attendees and their family members into the target population pool, the chance we pick up a child is higher than the chance to choose an adult. Because a child can be selected into the target pool either he/she is on travel or his/her family members is on travel, which is more likely to be selected than a single adult who can be chosen only when him/her on travel/attending mass gatherings. This explains the higher proportion of children in the target population as shown in Table S4.

Children are assumed to have higher susceptibility and infectiousness than adults and elderly [6], thus even

Table S5. Adjusted Peak Prevalence Value and Total Attack Rate.

Traveling/Mass Gathering Starting Time	Target Population	Peak Prevalence	Total Attack Rate
Day 30	Adults' Group	2.77%	52.3%
	Children's Group	3.09%	54.8%
	**Adjusted Value**	**2.86%**	**53.0%**
Day 60	Adults' Group	2.86%	52.7%
	Children's Group	3.27%	55.5%
	**Adjusted Value**	**2.97%**	**53.5%**
Day 90	Adults' Group	2.60%	52.3%
	Children's Group	3.05%	55.0%
	**Adjusted Value**	**2.73%**	**53.0%**
Baseline (no traveling/mass gatherings)	Adults' Group	2.60%	49.4%
	Children's Group	3.05%	53.8%
	**Adjusted Value**	**2.73%**	**51.0%**

in the baseline scenario (without traveling occurring), the peak prevalence level in the target pool (2.85%) is higher than the average peak prevalence level (2.73%) in the entire state. To isolate the impact of traveling, we obtain the total attack rate and the peak prevalence value in the children's group (the first three age groups) and in the adults' group (the last two age groups) separately for the target population.

The results are listed in Table S5. In the scenarios shown in Table S5, 10% of the population are assumed to be on travel/attending mass gatherings during a 1-day traveling/mass gatheirng period, and $R_0 = 1.5$. From Table S5, we can observe that in cases where traveling/mass gathering occurs before the epidemic peak (e.g., $t* = 30$ or 60), both the adult and children groups in the target population have higher peak prevalence value and total attack rate than in the baseline cases. Even when the traveling/mass gathering occurs after the peak (e.g., $t* = 90$), the adult and children groups in the target population have higher total attack rates than in the baseline cases.

The cesus data [10] shows that 72.29% of the population in Georgia is adults. Suppose the peak prevalence value we get is P_{child} in the children's group and P_{adult} in the adults' group, the adjusted peak prevanlence value P_{adjust} for the entire population will be the weighted average:

$$P_{adjust} = 0.2771 \cdot P_{child} + 0.7229 \cdot P_{adult}$$

The total attack rate can be adjusted in a similar way. Table S5 also shows the adjusted peak prevalence value and total attack rate in some of the experimented scenarios.

References

[1] Wu JT, et al. Reducing the impact of the next influenza pandemic using household-based public health interventions. PLoS Medicine 2006; 3: 1532-1540.

[2] Ferguson NM, et al. A population-dynamic model for evaluating the potential spread of drug-resistant influenza virus infections during community-based use of antivirals. Journal of Antimicrobial Chemotherapy 2003; 51: 977-990.

[3] Ferguson NM, et al. Strategies for mitigating an influenza pandemic. Nature 2006; 442: 448-452.

[4] Germann TC, et al. Mitigation strategies for pandemic influenza in the United States. Proceedings of the National Academy of Sciences USA 2006; 103: 5935-5940.

[5] Longini IM, et al. Containing pandemic influenza at the source. Science 2005; 309: 1083-1087.

[6] Carrat FJ, et al. A "small-world-like" model for comparing interventions aimed at preventing and controlling influenza pandemics. BMC Medicine 2006; 4(26).

[7] Ferguson NM, et al. Strategies for containing an emerging influenza pandemic in Southeast Asia. Nature 2005; 437: 209-214.

[8] Georgia Accrediting Commission. 2008 (http://www.coe.uga.edu/gac). Accessed 5 May 2008.

[9] Ekici A, Keskinocak P, Swann JL. Modelling Influenza Pandemic, Intervention Strategies, and Food Distribution, 2009. (http://www.isye.gatech.edu/pandemic/ modeling_panflu_EC). (Accessed June 15, 2009).

[10] Bureau of the Census. Census 2000. Washington, DC: Bureau of the Census, 2008. (http://www.census.gov). (Accessed May 1, 2008).

[11] Halder N, Kelso JK, Milne GJ: Analysis of the effectiveness of interventions used during the 2009 A/H1N1 influenza pandemic. BMC Public Health 2010, 10.

[12] Chin TD, et al. Morbidity and mortality characteristics of Asian strain influenza. Public Health Rep. 1960; 75: 149-158.

[13] Patel R, Longini IM, Halloran, E. Finding optimal vaccination strategies for pandemic influenza using genetic algorithms. Journal of Theoretical Biology 2005; 234: 201-212.

[14] Riley S. Large-scale spatial-transmission models of infectious disease. Science 2007;316(5829):1298-301.
